# The Keystone Veterinary Medical Association

**Published:** 1900-04

**Authors:** E. M. Ranck

**Affiliations:** Secretary pro tem.


Secretary.

### THE KEYSTONE VETERINARY MEDICAL ASSOCIATION.

The regular monthly meeting was held at Dental Hall, Thirteenth and Arch Streets, Tuesday, April 17, 1900, President Eves in the chair, who called the meeting to order at 9.15 P.M. Owing to the absence of Secretary Rhoads, the chair appointed E. M. Ranck, Secretary *pro tem.* The reading of the minutes of the previous meeting and roll-call had to be dispensed with on account of the absence of the Secretary and minute-books. The following members of the profession were present: Drs. Eves, Hoskins, Lintz, Pearson, Marshall, T. B. Rayner, and Mr. Carlisle, '01 Veterinary Department of the University of Pennsylvania.

Report of Room Committee, by C. J. Marshall, chairman, also presentation of bills for same, on which motion to pay respective amounts carried.

Discussion on "Milk" meeting, which was held in the same hall one month previous, by Drs. Hoskins, Marshall, and Eves, followed by remarks by Dr. Hoskins on the removal of Dr. J. W. Adams as consulting city veterinarian, as well as the resignation of one of the milk inspectors and the appointment of a milk gatherer to the same position by the Mayor at the same salary.

Discussion on the agitation of the "oleomargarine" and other food products brought out the fact that the subordinate officials seem to be well protected by the governing officials, the whole affair based on the "spoils system."

Report of a case of azoturia, by Dr. Hoskins, in a Western mare which had partially recovered from an attack of influenza, the peculiarity of the case being a paralysis of the nerve supplying the anterior straight muscle of one posterior limb. Treatment: Usual course in connection with electricity, with no marked effect.

There being no further business, the meeting adjourned to meet at the same place the third Tuesday in May.

E. M. Ranck,
Secretary pro tem.

